# Immunopharmacology of gastric cancer–deciphering immune cell subset responses and nanoparticle-mediated targeting

**DOI:** 10.3389/fphar.2025.1611234

**Published:** 2025-05-19

**Authors:** Yanting Wang, Ning Ding, Lina Qi, Wenwen Chen, Panyisha Wu

**Affiliations:** ^1^ Department of Gastroenterology, The Second Affiliated Hospital of Zhejiang University School of Medicine, Hangzhou, Zhejiang, China; ^2^ Department of Medical Oncology, The Second Affiliated Hospital of Zhejiang University School of Medicine, Hangzhou, Zhejiang, China; ^3^ School of Medicine, University of California, San Diego, San Diego, CA, United States

**Keywords:** gastric cancer, immune cell subsets, immunopharmacology, tumor microenvironment, targeted drug delivery, therapeutic resistance

## Abstract

The diverse landscape of immune cell populations significantly influences therapeutic outcomes in advanced gastric cancer, a leading cause of cancer mortality worldwide. Progress in immunopharmacology, aided by single-cell analytics, increasingly highlights immune complexity and functional heterogeneity. Conventional categories contain diverse subsets, including various T cells (helper, regulatory, memory) and B cells (plasma, memory, regulatory). Innate immune cells like macrophages, natural killer cells, and dendritic cells also exist in various functional states. These subsets exhibit distinct pharmacological response profiles that are often obscured by bulk analyses. This review explores the differential responses of critical immune cell subsets within the gastric cancer tumor microenvironment to current therapeutic modalities, encompassing cytotoxic chemotherapy, molecular targeted agents, and immunotherapies such as checkpoint inhibitors. We delve into the molecular processes underlying subset-specific drug effects, potential mechanisms of therapeutic resistance linked to specific immune cell states, and the influence of the tumor microenvironment on immune subset pharmacology. Furthermore, we discuss the application and potential of nanoparticle-based drug delivery systems specifically engineered to target distinct immune cell subpopulations, aiming to enhance immunomodulatory efficacy, reshape subset repertoires favorably, overcome resistance, and minimize toxicity for more precise and effective treatment of advanced gastric cancer.

## Introduction

The therapeutic landscape for complex diseases like cancer is increasingly shaped by our understanding of immunopharmacology, particularly how the diverse composition and functional states of immune cell populations within the tumor microenvironment (TME) critically influence treatment outcomes (Bejarano et al., 2021). Recent technological advances, especially in single-cell analytics, have unveiled profound heterogeneity within traditional immune cell classifications, revealing numerous subsets of T cells (e.g., CD4^+^, CD8^+^, helper T cells, regulatory T cells (Tregs), and memory T cells) (Zheng et al., 2021), B cells (e.g., plasma, memory, regulatory) (Fitzsimons et al., 2024), macrophages (e.g., M1/M2 polarization states), and other innate immune cells, each potentially exhibiting distinct pharmacological response profiles.

Gastric cancer, the fifth most diagnosed cancer and third-leading cause of cancer death globally (Sung et al., 2021), highlights these challenges, as most cases are diagnosed late despite screening efforts (Smyth et al., 2020), and survival drops significantly beyond early stages (Ajani et al., 2022). Despite advances in novel endoscopic techniques in diagnosing and treatment for early-stage gastric cancer in a minimally invasive way in recent years (Ji et al., 2024), most cases are diagnosed at later stages, due to that patients commonly experience no specific clinical symptoms in the early stage of gastric cancer. Tremendous efforts have been taken including public health policies and implementing screening programs to improve early detection and mortality.

Traditional cytotoxic chemotherapy continues to be mainstay of treatment to prolong the overall survival and progression-free survival in patients with advanced unresectable and metastatic gastric cancer. These agents include 5-fluorouracil (5-FU), fluoropyrimidine, platinum agents (cisplatin, oxaliplatin), taxanes (paclitaxel, docetaxel), and irinotecan, used as a single agent or in combination. Several clinical trials assessed the efficacy of various second-line agents when patients fail to respond to the first-line therapy regimens. However, lower response rates and more adverse drug reactions including fatigue, neuropathy, dermatologic toxicity, immune depression, myelosuppression and many others were demonstrated, stemming from their nonspecific nature.

Novel molecular targeted agents, such as the anti-HER2 antibody trastuzumab (Zhang et al., 2025), and immune checkpoint inhibitors have shown benefit in subsets of patients (Scheck et al., 2024), but their efficacy is influenced by the TME. Other targeted agents that attracted a great deal of interest from researchers include EGFR antagonists, VEGF Inhibitors, EGFR and HER2 tyrosine kinases dual inhibitor, PI3K/Akt/mTOR inhibitor, matrix metalloproteinase (MMP) inhibitors, HGF/c-MET inhibitors and more recently, promising immune checkpoint inhibitors including cytotoxic T-lymphocyte-associated protein 4 (CTLA-4) inhibitor and programmed death protein 1/programmed death-ligand 1 (PD-1/PD-L1) inhibitors.

The efficacy, resistance patterns, and side effects of these diverse treatments are increasingly recognized as being influenced by their differential impact on specific immune cell subsets within the TME. Understanding how therapies distinctively affect populations such as Tregs, various polarized tumor-associated macrophages (TAMs), myeloid-derived suppressor cells (MDSCs), effector T cells, natural killer (NK) cells, and B cell subsets is critical for developing more effective strategies.

Precision oncology is advancing via nanotechnology (Linderman et al., 2025), using nanoparticles with tunable properties to improve drug delivery. While initially focused on tumor targeting, engineering nanoparticles to modulate specific immune subpopulations within the TME is an emerging approach (Jin et al., 2024) holding potential for enhancing efficacy and overcoming resistance in advanced gastric cancer.

Therefore, this review aims to synthesize recent progress in advanced gastric cancer therapies, specifically examining their interactions with diverse immune cell subsets and the implications of these subset-specific pharmacological responses. We will explore the molecular mechanisms governing these differential effects and discuss the burgeoning application of nanoparticle-based drug delivery systems as a novel strategy to precisely target key immune cell subpopulations, ultimately seeking to enhance the efficacy of immunomodulatory treatments for gastric cancer.

## The diverse immune landscape in the gastric cancer tumor

The initiation, progression, and therapeutic response of gastric cancer are profoundly influenced by the complex interplay between malignant cells and their surrounding TME. Far from being a passive bystander, the TME is a dynamic ecosystem comprising not only cancer cells but also a diverse array of nonmalignant stromal cells, extracellular matrix components, and crucially, a heterogeneous infiltrate of immune cells, all engaged in intricate signaling networks. It is now well-recognized that the TME plays a vital role in oncogenesis, often dampening anti-tumor immune responses and significantly affecting the efficacy of various cancer therapies (Pitt et al., 2016).

This complexity is clearly manifested in gastric cancer, a major global health burden. Understanding the specific composition and functional states of the immune cell populations within the gastric TME is therefore paramount for developing next-generation treatments. Specific features of the gastric cancer TME significantly shape its immune landscape and clinical behavior. For instance, high infiltration of FoxP3+ Tregs is frequently observed, correlating with advanced disease and poorer prognosis (Negura et al., 2023; Shen et al., 2010). The immune contexture also varies distinctly with gastric cancer molecular subtypes; microsatellite instability-high (MSI-H) tumors, for example, typically harbor higher densities of CD8^+^ TILs, potentially underlying their better response to checkpoint inhibitors (Petrillo et al., 2025). Furthermore, etiological factors like chronic *Helicobacter pylori* infection critically shape the initial gastric inflammatory milieu and subsequent immune composition long before overt malignancy develops. *H. pylori* employs virulence factors, such as urease for survival and the CagA oncoprotein injected via a type IV secretion system, to persist within the gastric mucosa despite host immune responses (Mohammadzadeh et al., 2023). This persistence drives chronic inflammation, characterized by the recruitment and activation of various immune cells, including neutrophils, macrophages, and lymphocytes. The infection skews the immune response, often leading to an increase in immunosuppressive populations like Tregs and myeloid-derived suppressor cells (MDSCs), while potentially impairing the function of DCs and effector T cells (Baj et al., 2020). Macrophages recruited to the site often polarize towards a pro-tumor M2-like phenotype (TAMs), contributing to immune evasion, angiogenesis, and tissue remodeling. *H. pylori* also triggers the release of a complex array of cytokines from both immune and epithelial cells, including pro-inflammatory mediators like IL-1β, IL-6, IL-8, IL-17, IL-32, and TNF-α, which sustain inflammation, alongside immunosuppressive cytokines like IL-10 and TGF-β that can dampen effective anti-bacterial immunity and promote tolerance (Della Bella et al., 2023). This sustained, intricate inflammatory environment orchestrated by chronic *H. pylori* infection can contribute to DNA damage, genetic instability, and altered cell signaling pathways (e.g., NF-κB, STAT3) within gastric epithelial cells, ultimately creating a microenvironment conducive to carcinogenesis (Ding et al., 2010).

The adaptive immune system constitutes a critical component of the gastric TME infiltrate. CD8^+^ CTLs are central players in anti-tumor immunity due to their capacity to directly kill tumor cells (Sathe et al., 2020). Indeed, studies have shown that patients with high CD8^+^ tumor-infiltrating lymphocyte (TIL) counts often have significantly longer overall survival in gastric cancer (Pernot et al., 2020). However, the mere presence of CD8^+^ T cells is not sufficient; their functional state is critical. Within the TME, CD8^+^ T cells exist across a spectrum of differentiation states, including naive, effector, memory and critically, exhausted T cells (Tex). Menory cells including central memory (TCM), effector memory (TEM) and resident memory (TRM) T cells. T cell exhaustion, characterized by progressive loss of effector function and sustained expression of inhibitory receptors like PD-1, CTLA-4, TIM-3, Lag-3, and TIGIT, is a major mechanism of immune evasion in gastric cancer (Jiang et al., 2020). The interaction between PD-1 on T cells and its ligand PD-L1, often overexpressed on cancer cells or other TME cells, directly leads to T cell apoptosis or functional silencing, providing the rationale for checkpoint inhibitor therapies (Karim et al., 2023).

CD4^+^ T helper (Th) cells also display significant heterogeneity and plasticity within the gastric TME. While traditionally categorized into Th1 (promoting cell-mediated immunity), Th2 (driving humoral immunity), and Th17 (involved in inflammation and autoimmunity) subsets, their roles in cancer are context-dependent (Basu et al., 2021). Th1 cells, for instance, are generally considered anti-tumorigenic, aiding CTL responses, whereas Th2 and Th17 responses can sometimes promote tumor growth or inflammation that supports malignancy (Anvar et al., 2024). A particularly crucial CD4^+^ subset in the TME is the Treg (Negura et al., 2023), typically characterized by FoxP3 expression. Tregs are potent immunosuppressors, dampening anti-tumor responses mediated by CTLs and other effector cells. High infiltration of Tregs in the gastric TME is frequently associated with poor prognosis and resistance to immunotherapy. The balance between effector T cells and Tregs is thus a key determinant of the local immune status (Nishikawa and Koyama, 2021).

B lymphocytes, another arm of the adaptive immune system, also exhibit diverse roles within the gastric TME (Wei et al., 2021). Beyond their classical function of producing antibodies, B cells can differentiate into plasma cells, long-lived memory B cells, or function as antigen-presenting cells. Furthermore, specific subsets like regulatory B cells (B regs) have been identified, which, similar to Tregs, exert immunosuppressive functions, potentially hindering anti-tumor immunity (Xue et al., 2024). The organization of B cells, T cells, and DCs into tertiary lymphoid structures (TLS) within or near the tumor site has also gained attention (Sautès-Fridman et al., 2019), as the presence and maturity of TLS can correlate with better prognosis and response to immunotherapy in some cancers (Cabrita et al., 2020; Schumacher and Thommen, 2022), although their precise role in gastric cancer requires further clarification.

The innate immune system also populates the gastric TME with functionally diverse cells. TAMs are often the most abundant immune cell type (Bied et al., 2023), arising from recruited monocytes that differentiate under the influence of local TME signals (Pan et al., 2020). While historically simplified into a dichotomy of anti-tumor M1 and pro-tumor M2 phenotypes, it is now clear that TAM polarization represents a spectrum of activation states with considerable plasticity (Qin et al., 2020; Ricketts et al., 2021). Pro-tumor M2-like TAMs contribute significantly to gastric cancer progression by promoting angiogenesis, matrix remodeling, tumor cell invasion, and suppressing adaptive immunity (Zhao et al., 2021). Targeting TAMs has therefore become an attractive therapeutic strategy. This includes blocking “do not eat me” signals like CD47-SIRPα or CD24-Siglec-10 to enhance phagocytosis by macrophages, inhibiting monocyte recruitment or differentiation via pathways like CSF-1/CSF-1R, or attempting to repolarize M2-like TAMs towards an anti-tumor M1-like state.

Other myeloid cells, such as dendritic cells (DCs) and MDSC, are also critical regulators of the gastric TME (Wang et al., 2024). DCs, including conventional DC subsets (cDC1, cDC2) and plasmacytoid DCs (pDCs), are professional antigen-presenting cells essential for initiating anti-tumor T cell responses. However, DCs within the TME are often functionally impaired or skewed towards tolerogenic phenotypes, contributing to immune evasion (Xiao et al., 2023). MDSCs, encompassing monocytic (M-MDSC) and polymorphonuclear (PMN-MDSC) subsets, are potent immunosuppressive cells that accumulate in gastric cancer patients and inhibit T cell and NK cells function through various mechanisms, representing another major barrier to effective anti-tumor immunity (Zhang Y. et al., 2024).

Innate lymphoid cells, particularly NK cells, contribute to immune surveillance in the gastric TME (Terrén et al., 2019). NK cells can directly lyse tumor cells without prior sensitization. However, similar to T cells, NK cells’ function is often suppressed within the TME through inhibitory receptor engagement or exposure to suppressive factors, limiting their anti-cancer activity. Different NK cell subsets may also possess distinct functional capacities and susceptibilities to TME-mediated inhibition (Meza et al., 2020).

Therefore, the gastric cancer TME is characterized by a complex and highly heterogeneous immune infiltrate. The specific composition, density, spatial organization, and functional polarization of diverse immune cell subsets–ranging from effector and regulatory lymphocytes to varied myeloid populations–collectively shape the local immunological context. This intricate immune landscape not only dictates the natural course of the disease but also critically influences the efficacy and potential toxicity of virtually all systemic gastric cancer treatments, including traditional cytotoxic chemotherapy, molecular targeted therapies, and immunotherapies. Understanding the differential pharmacological responses of these specific immune cell subsets to therapy is therefore essential for moving beyond broad treatment approaches towards more precise and effective immunopharmacological interventions.

## Differential pharmacological responses of immune cell subsets to gastric cancer therapies

The treatment armamentarium for advanced gastric cancer includes cytotoxic chemotherapy, molecularly targeted agents, and immunotherapies, often used sequentially or in combination. While traditionally evaluated primarily for their direct effects on tumor cells, it is now unequivocally clear that these therapies exert profound and often differential effects on the diverse immune cell subsets residing within or trafficking through the TME. These immunomodulatory consequences significantly influence not only therapeutic efficacy but also resistance mechanisms and adverse events. Understanding the distinct pharmacological responses of specific immune cell subpopulations, ranging from effector and regulatory lymphocytes to myeloid cells, to each class of gastric cancer therapy is therefore critical for optimizing treatment strategies and developing novel immunopharmacological approaches.

### Impact of cytotoxic chemotherapy on immune subsets

Traditional cytotoxic chemotherapy remains a cornerstone for treating advanced unresectable and metastatic gastric cancer. Commonly used agents include fluoropyrimidines (like 5-fluorouracil), platinum agents (cisplatin, oxaliplatin), taxanes (paclitaxel, docetaxel), and irinotecan, employed either as single agents or, more frequently, in combination regimens. While effective in prolonging survival for some patients, their utility is often limited by significant adverse drug reactions, including fatigue, neuropathy, dermatologic toxicity, and notably, myelosuppression and general immune depression, stemming largely from their non-specific targeting of rapidly dividing cells.

Beyond broad immunosuppression, however, chemotherapeutic agents can induce complex and often drug-specific alterations in the composition and function of distinct immune cell subsets (Galluzzi et al., 2015; Mukherjee et al., 2023). Many chemotherapies cause lymphodepletion, but the sensitivity varies among lymphocyte populations. Highly proliferative effector T cells (Teffs) can be susceptible, but Tregs, which often proliferate within the TME to maintain suppression, can also be depleted by certain agents (e.g., cyclophosphamide, though less commonly used in GC first-line) (Cai et al., 2024). Some agents, like 5-FU or oxaliplatin, have been reported to selectively deplete MDSCs, potent inhibitors of anti-tumor immunity (Kim and Kim, 2019; Vincent et al., 2010). Taxanes might interfere with Treg function or promote DC maturation. Thus, depending on the specific agent, dose, and schedule, chemotherapy can paradoxically alleviate immunosuppression by targeting suppressive subsets like Tregs or MDSCs more effectively than effector cells.

Furthermore, certain chemotherapies (e.g., anthracyclines, oxaliplatin) can induce immunogenic cell death (ICD) in tumor cells (Wang et al., 2018). ICD involves the release of damage-associated molecular patterns (DAMPs) and tumor-associated antigens (Inoue and Tani, 2014), which can promote DC maturation, enhance antigen cross-presentation to CD8^+^ T cells, and ultimately stimulate adaptive anti-tumor immunity. This interplay highlights that chemotherapy’s impact extends beyond direct cytotoxicity to actively modulating innate and adaptive immune responses, often in a subset-dependent manner. The overall immunological outcome—whether net immunosuppression or immune activation—likely depends on the specific drug, the baseline immune context of the patient’s TME, and the intricate balance of effects on various pro- and anti-tumor immune subsets. Understanding these differential effects is crucial for rationally combining chemotherapy with immunotherapy.

### Impact of molecular targeted therapies on immune subsets

The development of molecular targeted therapies, aimed at specific oncogenic drivers, represented a significant advance. In gastric cancer, key examples include HER2-targeted, EGFR-targeted, and VEGF/VEGFR-targeted agents (Zeng and Jin, 2022). While designed to act primarily on tumor cells expressing the target molecule, these therapies also have important, though less completely understood, interactions with the immune system, often mediated through specific immune cell subsets.

HER2-targeted therapy, primarily with the monoclonal antibody trastuzumab, is standard for the subset of gastric cancer patients with HER2 overexpression (Shao et al., 2025). Trastuzumab not only blocks HER2 signaling but also crucially induces antibody-dependent cellular cytotoxicity (ADCC). This immune-mediated mechanism relies heavily on effector cells expressing Fc receptors (FcγRs), primarily NK cells and, to some extent, macrophages (Grandits et al., 2025). The efficacy of trastuzumab is therefore likely influenced by the abundance, activation state, and specific phenotype (e.g., FcγR polymorphisms) of NK cell subsets and potentially certain macrophage subsets within the TME. Conversely, resistance to trastuzumab, which frequently develops, might involve mechanisms that impair ADCC, such as downregulation of HER2, shedding of the target, or alterations in the TME that suppress NK cell function. While other HER2-targeted agents like pertuzumab, lapatinib, and T-DM1 have shown limited success in later-line gastric cancer trials, the newer antibody-drug conjugate trastuzumab deruxtecan has shown promising activity, potentially due to its potent payload and bystander effect, which might also interact differently with the immune milieu (Riccardi et al., 2023; Yu et al., 2024).

EGFR-targeted therapies, such as cetuximab, have yielded disappointing results in unselected advanced gastric cancer patients, although their potential role in strictly defined EGFR-amplified subgroups is still being explored. Similar to anti-HER2 antibodies, anti-EGFR antibodies can mediate ADCC, suggesting that the presence and functional state of NK cell subsets could influence response in susceptible tumors (Maron et al., 2018).

Anti-angiogenic therapies targeting the VEGF pathway (e.g., the anti-VEGFR-2 antibody ramucirumab, or TKIs like apatinib, regorafenib have demonstrated benefit in later-line settings. Beyond inhibiting tumor neovascularization, VEGF signaling profoundly impacts the immune TME. VEGF promotes the accumulation and function of immunosuppressive Tregs and MDSCs, impairs DC maturation and antigen presentation, and can directly inhibit T cell activity (Ribatti, 2022). Anti-VEGF therapies can counteract these effects: they may reduce Treg and MDSC infiltration, promote DC maturation, and “normalize” the tumor vasculature, potentially facilitating the infiltration and function of effector T cells (Fricke et al., 2007). Therefore, the clinical benefit derived from anti-angiogenic agents likely involves modulation of these specific immune cell subsets, contributing to a less immunosuppressive TME.

### Impact of immunotherapies on immune cell subsets

Immunotherapy, particularly immune checkpoint inhibition (CPI), has revolutionized cancer treatment, including offering new hope for a subset of advanced gastric cancer patients. These therapies primarily function by modulating the activity of specific immune cell subsets. Anti-PD-1/PD-L1 antibodies (e.g., pembrolizumab, nivolumab) work by disrupting the interaction between the inhibitory receptor PD-1, highly expressed on exhausted T cells, Tregs, and other immune cells, and its ligand PD-L1, often upregulated on tumor cells and antigen-presenting cells (Han et al., 2020). Blocking this axis primarily aims to reinvigorate pre-existing, tumor-reactive CD8^+^ T cells that have become dysfunctional or “exhausted” within the TME. The response to anti-PD-1/PD-L1 therapy is therefore highly dependent on the presence and state of specific T cell subsets. Patients whose tumors are infiltrated by CD8^+^ T cells expressing PD-1, but which are not terminally exhausted, are more likely to benefit. Predictive biomarkers such as PD-L1 expression (on tumor or immune cells), high microsatellite instability (MSI-H) or mismatch repair deficiency (dMMR), and high tumor mutational burden (TMB) are surrogates for an inflamed TME likely containing neoantigens and potentially reactive, albeit suppressed, T cell populations (Ma et al., 2022). Anti-PD-1/PD-L1 therapy can also impact other subsets; it may decrease the suppressive capacity of Tregs (which also express PD-1), potentially enhance NK cell activity, and modulate macrophage function. Pembrolizumab has shown efficacy in previously treated, PD-L1-positive advanced gastric cancer and is recommended in later-line settings (Bai and Cui, 2022).

Anti-CTLA-4 antibodies target a different checkpoint, primarily acting earlier during T cell priming in lymphoid organs, potentially leading to broader T cell activation and proliferation but also increased immune-related adverse events (Wei et al., 2018). Combination strategies targeting both PD-1 and CTLA-4 are under investigation.

Beyond CPIs, other immunotherapeutic approaches target different immune cell interactions. Chimeric antigen receptor (CAR)-T cell therapy, while highly successful in hematological malignancies, faces challenges in solid tumors like gastric cancer due to antigen heterogeneity, trafficking difficulties, and the suppressive TME (Wagner et al., 2020). Its success hinges on overcoming suppressive subsets like Tregs and MDSCs within the gastric TME. Macrophage-directed therapies are also gaining traction. Strategies include blocking “do not eat me” signals like CD47 (recognized by SIRPα on macrophages) or CD24 (recognized by Siglec-10) to unleash macrophage phagocytosis of tumor cells, inhibiting the recruitment of pro-tumor monocytes using CSF-1R inhibitors, or attempting to repolarize pro-tumor M2-like TAMs towards an anti-tumor M1-like phenotype using various agents (Li et al., 2020). Each of these approaches relies on understanding and manipulating the specific biology and pharmacological responsiveness of distinct macrophage subsets or their precursors.

The clinical activity of all major systemic therapies for advanced gastric cancer—cytotoxic chemotherapy, targeted agents, and immunotherapies—is intricately linked to their complex and differential interactions with the diverse array of immune cell subsets within the TME. Recognizing that each therapy can uniquely sculpt the immune landscape, affecting the balance between effector and suppressor populations, is crucial. This nuanced, subset-focused perspective on immunopharmacology provides the foundation for designing more rational combination therapies and developing innovative strategies, such as subset-specific targeting, to overcome resistance, enhance efficacy, and ultimately improve outcomes for patients with this challenging malignancy.

## Mechanisms of immune subset-mediated therapeutic resistance in gastric cancer

Despite advances in systemic therapies, therapeutic resistance remains a major obstacle in managing advanced gastric cancer, limiting the duration and effectiveness of treatment and contributing to poor patient outcomes (Luo et al., 2025). Resistance can be primary (intrinsic lack of response) or acquired (developing after an initial response) and arises from complex interactions involving tumor cell-intrinsic factors and the dynamic TME. Increasingly, the composition, functional state, and spatial organization of diverse immune cell subsets within the TME are recognized as critical mediators of resistance to cytotoxic chemotherapy, molecularly targeted agents, and especially immunotherapies (Lei et al., 2023). Elucidating these immune subset-mediated resistance mechanisms is crucial for developing strategies to overcome therapeutic failure.

### Role of regulatory and suppressor immune subsets in therapeutic resistance

A key mechanism by which the TME fosters resistance is through the accumulation and activation of immunosuppressive immune cell populations that actively dampen anti-tumor immunity required for the efficacy of many treatments (Czajka-Francuz et al., 2023).

#### Tregs

High infiltration of Tregs into the gastric TME is often associated with advanced disease and poor prognosis (Wang et al., 2023). Tregs employ multiple suppressive mechanisms, including the secretion of inhibitory cytokines like IL-10 and TGF-β, expression of inhibitory receptors like CTLA-4, consumption of the T cell growth factor IL-2, and metabolic disruption of effector cells. This potent immunosuppressive activity can directly counteract the intended effects of immune checkpoint inhibitors (CPIs) by maintaining suppression even when pathways like PD-1 are blocked. Furthermore, some conventional therapies, including certain chemotherapies or targeted agents, might inadvertently spare or even promote the relative expansion or functional enhancement of Tregs, contributing to acquired resistance (Kumagai et al., 2024). Targeting Tregs, either through depletion (e.g., via anti-CCR4 antibodies or low-dose cyclophosphamide) or functional modulation (e.g., targeting CTLA-4 or specific Treg metabolic pathways), is an active area of investigation to overcome resistance.

#### TAMs

The M2-like TAMs are key architects of therapeutic resistance through diverse mechanisms (Jayasingam et al., 2019). These TAMs employ diverse resistance mechanisms. They secrete immunosuppressive factors (e.g., IL-10, TGF-β) and express checkpoint ligands (e.g., PD-L1) that inhibit T cells. Additionally, they promote angiogenesis, potentially limiting drug delivery and causing resistance to anti-VEGF therapy. TAMs also remodel the extracellular matrix, creating physical barriers, and directly support tumor cell survival and invasion (Di Ceglie et al., 2024). They can even metabolize certain chemotherapeutic drugs, reducing their local concentration. Overcoming TAM-mediated resistance involves strategies targeting their recruitment (e.g., CCL2/CCR2 blockade), survival/differentiation (e.g., CSF-1/CSF-1R inhibition), function (e.g., blocking “do not eat me” signals like CD47), or attempting to repolarize them towards an anti-tumor M1-like state (Li et al., 2023).

#### MDSCs

MDSCs, comprising monocytic (M-MDSC) and polymorphonuclear (PMN-MDSC) subsets, are immature myeloid cells with potent immunosuppressive capabilities. The broad immunosuppression significantly contributes to primary and acquired resistance, particularly against immunotherapies like CPIs (Li et al., 2021). Targeting MDSCs, either by inhibiting their development/recruitment, blocking their suppressive functions (e.g., inhibiting ARG1 or iNOS), or promoting their differentiation into mature, non-suppressive myeloid cells, is another strategy being explored to overcome resistance (Gao et al., 2020).

### Role of effector immune subset dysfunction in therapeutic resistance

Resistance can also arise from the failure or dysfunction of effector immune cells intended to mediate tumor destruction.

#### T cell exhaustion

While CPIs aim to reinvigorate exhausted T cells (Tex), resistance often occurs when T cell exhaustion is too profound or epigenetically “fixed.” Tex cells progressively lose effector functions (cytokine production, proliferation, cytotoxicity) and upregulate multiple inhibitory receptors (PD-1, TIM-3, Lag-3, etc.) (Chow et al., 2022). If tumor-specific T cells within the gastric TME are terminally differentiated or lack the capacity for functional restoration upon checkpoint blockade, CPI therapy will fail (primary resistance) (Said and Ibrahim, 2023). Furthermore, chronic antigen exposure and suppressive signals during therapy can potentially drive initially responsive T cells towards deeper exhaustion states, contributing to acquired resistance. The specific phenotype and reversibility of T cell exhaustion within different subsets (e.g., progenitor exhausted vs. terminally exhausted) likely dictates CPI sensitivity.

#### NK cell dysfunction

Resistance to HER2-targeted therapies like trastuzumab in gastric cancer is frequently linked to impaired ADCC. Studies specifically in GC patients indicate that dysfunction or low baseline activity of NK cells, the primary mediators of ADCC, correlates with poor response and shorter survival times following trastuzumab treatment (Wu et al., 2020). Additionally, acquired resistance can emerge through mechanisms like the downregulation of HER2 expression on tumor cells, reducing the target available for trastuzumab binding and subsequent ADCC.

### Contribution of the broader TME and therapy-induced evolution

The interplay between immune subsets and other TME components, like cancer-associated fibroblasts (CAFs), further complicates resistance. While not immune cells themselves, CAFs are abundant in the gastric TME stroma and are critical mediators of therapeutic resistance (Sun et al., 2022). They contribute to immune evasion and resistance by modulating immune subsets. CAFs secrete a wide array of factors, including IL-6, IL-11, TGF-β, and various chemokines, that can recruit and activate immunosuppressive Tregs and MDSCs, skew TAM polarization towards the M2 phenotype, directly inhibit effector T cell function, and promote angiogenesis (Mao et al., 2021). CAFs also extensively remodel the extracellular matrix, creating dense stromal barriers that physically impede immune cell infiltration and function. CAF-derived extracellular vesicles containing factors like Annexin A6 can also confer drug resistance (Piper et al., 2020). Thus, CAFs act as orchestrators of an immunosuppressive and therapy-resistant niche by influencing the behavior of key immune cell subsets.

Finally, acquired resistance often involves dynamic changes in the tumor and its microenvironment under therapeutic pressure. Treatments can select for tumor cell clones with reduced immunogenicity (e.g., loss of antigen presentation machinery) or intrinsic resistance mutations. Therapies can also reshape the immune landscape itself, for instance, by upregulating alternative immune checkpoints on T cells following PD-1 blockade (leading to secondary resistance) or by enriching populations of resistant Tregs or MDSCs (Cui et al., 2024).

Therefore, therapeutic resistance in advanced gastric cancer is a multifaceted problem significantly driven by the complex interactions within the TME, particularly involving the diverse array of immune cell subsets. Suppressive populations like Tregs, M2-like TAMs, and MDSCs actively curtail anti-tumor immunity and promote resistance, while dysfunction or exhaustion of effector cells like CD8^+^ T cells and NK cells limits therapeutic efficacy. Understanding the specific roles these subsets play in resistance to chemotherapy, targeted therapy, and immunotherapy is paramount. Overcoming resistance will likely require combination strategies that not only target the tumor directly but also reprogram the TME by modulating the function, composition, and interactions of key immune cell subpopulations.

## Nanoparticle-based strategies for targeting specific immune cell subsets in gastric cancer

The inherent limitations of conventional systemic cancer therapies, including suboptimal efficacy, dose-limiting toxicities often impacting the immune system, and the pervasive challenge of therapeutic resistance frequently orchestrated by the TME, have catalyzed the development of sophisticated drug delivery platforms (Vincent et al., 2022). Nanomedicine, which utilizes materials engineered at the nanoscale (typically 1–1,000 nm), represents a rapidly advancing Frontier with transformative potential for oncology. Nanoparticles (NPs) offer a versatile toolkit, possessing unique physicochemical characteristics, precisely controllable size and surface functionalities, and the capacity to encapsulate a wide spectrum of therapeutic payloads, from small molecule drugs to large biologics like proteins and nucleic acids (Chehelgerdi et al., 2023). These attributes can significantly enhance therapeutic outcomes by improving drug solubility, protecting cargo from premature degradation, modifying pharmacokinetic profiles, and enabling targeted delivery. Consequently, NPs can lead to improved specific biodistribution, and bioavailability compared to conventional unmodified drugs (Jarmila et al., 2024).

Early nanomedicine strategies often relied on passive accumulation in tumors via the enhanced permeability and retention (EPR) effect or active targeting of receptors highly expressed on cancer cells. While valuable, a more nuanced and potentially powerful approach, aligning directly with the aims of precision immunopharmacology, involves the rational design of nanoparticle systems to specifically target and functionally modulate distinct immune cell subpopulations within the complex gastric TME (Golombek et al., 2018; Shi et al., 2020). This strategy involves concentrating therapeutics (e.g., immunomodulators, cytotoxic agents) within specific immune cells. Targets include key effector cells (like CTLs, NK cells, DCs) or immunosuppressive populations (such as Tregs, TAMs, MDSCs). The goal is to reshape the immune landscape favorably, overcome resistance, and enhance anti-tumor efficacy while minimizing systemic side effects (Zhu et al., 2020; Peng et al., 2022). Although dedicated investigation of such targeted nano-immunotherapies specifically in gastric cancer remains somewhat limited compared to other tumor types, the fundamental principles and rapidly evolving technologies offer considerable promise for this challenging malignancy.

The rationale for employing nanoparticles to target specific immune subsets is multifaceted. Firstly, it addresses the critical need for spatial control in immunomodulation. Systemic administration of potent immune agonists or antagonists often leads to widespread, off-target effects and severe toxicities (e.g., cytokine release syndrome, autoimmunity). By concentrating these agents within specific immune cell populations residing in or trafficking to the TME, NPs can potentially maximize local therapeutic impact while minimizing systemic exposure (Gao et al., 2019). Secondly, targeting specific subsets allows for tailored functional modulation. For instance, NPs can be designed to deliver activating signals (e.g., cytokines, TLR agonists) to effector cells like CTLs or DCs, or inhibitory signals/payloads (e.g., cytotoxic drugs, metabolic inhibitors, siRNA) to suppressive cells like Tregs or M2-like TAMs. Thirdly, nanoparticles can overcome biological barriers, protect labile cargos (like peptides or nucleic acids) en route to the target cell, and facilitate cellular uptake, which can be inefficient for free drugs or biologics (Chen et al., 2023). Lastly, NPs serve as ideal platforms for co-delivering multiple agents (e.g., a chemo-drug and an immune modulator, or multiple immunomodulators) to the same immune cell, enabling synergistic therapeutic effects that are difficult to achieve with combinations of free drugs possessing different pharmacokinetic profiles (Li et al., 2024).

Diverse nanoparticle platforms are utilized for immunomodulatory applications. Biocompatible lipid-based systems, such as liposomes and nanostructured lipid carriers (NLCs), are widely used for encapsulating various drugs, with some NLC modifications showing potential in gastric cancer models (Singh et al., 2025). Lipid nanoparticles (LNPs) are particularly prominent for delivering nucleic acids, enabling strategies like siRNA-mediated gene silencing in immune cells (Kuznetsova et al., 2024). Polymeric systems offer tunable drug release and stability, while inorganic nanoparticles (e.g., gold or iron oxide) provide unique properties for imaging or localized therapies, which can be combined with immunomodulation (Zhang W. et al., 2024).

For DCs, NPs are designed to enhance antigen presentation by co-delivering tumor antigens and potent adjuvants (like TLR agonists or STING agonists) using DC-targeting ligands (anti-CD11c, anti-DEC205) or exploiting natural DC uptake mechanisms, potentially within formulations that promote cytosolic antigen release for cross-presentation (Chesson and Zloza, 2017). Regarding T lymphocytes, strategies focus on either inhibiting Tregs (via targeted delivery of cytotoxic drugs, mTOR inhibitors, or agents disrupting FoxP3 stability using markers like CD25 or CCR4) or boosting Teffs (via targeted delivery of IL-2, IL-12, IL-15, co-stimulatory molecules, or metabolic enhancers). Nanoparticle delivery of checkpoint inhibitors, either antibodies like anti-PD-L1 conjugates or small molecules, aims to enhance TME penetration and potentially reduce systemic immune-related adverse events compared to systemic administration (Liu et al., 2023). Targeting MDSCs often involves NPs delivering differentiation agents or inhibitors of their suppressive functions, potentially using markers like CD11b (Yang and Sun, 2024). Enhancing NK cell activity can be pursued with NPs delivering activating cytokines like IL-15 or by designing NPs to augment ADCC mediated by therapeutic antibodies like Trastuzumab.

The true power of NP-based immune subset targeting may lie in enabling complex combination therapies. NPs can co-deliver chemotherapy or targeted therapy agents along with immune modulators tailored to specific subsets, potentially overcoming resistance mechanisms and achieving synergy. For instance, combining NP-delivered chemotherapy (enhancing antigen release via ICD) with NP-delivered DC activators could potently boost anti-tumor T cell responses (Vincent et al., 2022). Similarly, combining Treg depletion NPs with subsequent CPI therapy could unlock responses in previously resistant tumors. Even targeting non-immune TME components like CAFs with NPs might be viewed through an immunopharmacological lens if the goal is to disrupt CAF-mediated immune suppression (e.g., reducing suppressive cytokine secretion or matrix barriers) to indirectly enhance immune effector cell function or infiltration.

Despite the exciting potential, significant challenges remain for the clinical translation of these sophisticated nanostrategies in gastric cancer. Achieving robust, specific, and efficient delivery to the intended immune subset within the heterogeneous and often poorly perfused human gastric TME is a major hurdle. Overcoming biological barriers, minimizing off-target accumulation, ensuring predictable drug release, addressing potential immunogenicity of the NPs themselves, and developing scalable, cost-effective manufacturing processes are all critical considerations. Furthermore, the dynamic nature and phenotypic plasticity of immune subsets complicate target selection and durability of response. Future directions will likely focus on developing “smart” stimuli-responsive nanoparticles that activate or release their payload specifically within the TME, creating multi-stage delivery systems, employing advanced computational modeling to predict NP behavior, and integrating theranostic capabilities for real-time monitoring. Ultimately, leveraging deep immune profiling of individual patients’ tumors using single-cell and spatial technologies will be key to identifying the most relevant subset targets and tailoring personalized nano-immunotherapy strategies.

In conclusion, nanoparticle-based drug delivery systems engineered to specifically target and modulate distinct immune cell subpopulations represent a highly promising Frontier in gastric cancer therapy. By moving beyond broad systemic effects or simple tumor targeting, these strategies offer the potential for unprecedented precision in manipulating the complex TME immune landscape. Successfully delivering activating signals to effector cells while neutralizing suppressive populations holds the key to overcoming resistance, enhancing the efficacy of immunotherapies and other treatments, and minimizing debilitating toxicities. While significant challenges remain in clinical translation, continued innovation in nanotechnology and immunopharmacology positions immune subset-targeted nanomedicine as a critical pathway toward more effective and personalized treatments for patients suffering from advanced gastric cancer.

## Future perspectives and conclusion

Advanced gastric cancer treatment faces challenges like resistance and toxicity. This review examined therapies through an immunopharmacological lens, highlighting how differential responses of diverse immune subsets within the TME impact outcomes from chemotherapy, targeted agents (like anti-HER2 therapy relying on NK cells), and checkpoint inhibitors (dependent on T cell status). We underscored the role of suppressive subsets (Tregs, TAMs, MDSCs) in resistance and explored nanomedicine’s potential for precisely targeting these populations.

Our discussion highlighted the complex, often dual-edged, ways conventional chemotherapies modulate the immune landscape beyond simple immunosuppression, the reliance of targeted therapies like anti-HER2 antibodies on specific immune effector subsets (e.g., NK cells for ADCC), and the central role of T cell subset status (e.g., exhaustion vs. activation) in dictating the success or failure of immune checkpoint inhibitors. Furthermore, we underscored the critical contribution of various immunosuppressive subsets, including Tregs, M2-like TAMs, and MDSCs, in orchestrating both primary and acquired resistance to multiple therapeutic modalities. Finally, we explored the burgeoning field of nanomedicine, not merely as a general drug delivery enhancement tool, but specifically as a platform for the precise targeting and functional modulation of these key immune cell subpopulations, a strategy holding immense promise but still in relatively early stages for gastric cancer compared to other malignancies.

Despite this progress, significant knowledge gaps remain, paving the way for exciting future research directions. A deeper, higher resolution understanding of the immune contexture specifically within human gastric cancer is urgently needed. This requires moving beyond simplistic classifications (e.g., M1/M2 TAMs) to comprehensively map the full spectrum of immune subset diversity, functional states, and spatial organization across different molecular subtypes of gastric cancer (e.g., EBV-positive, MSI-high, genomically stable, chromosomally unstable), stages of disease progression, and in response to various treatments. Critically, understanding the dynamic evolution of the immune landscape during therapy is essential for deciphering mechanisms of acquired resistance and identifying optimal windows for combination interventions. How do specific treatments reshape the balance between effector and suppressor subsets over time? What are the epigenetic or metabolic programs that sustain suppressive phenotypes or drive terminal T cell exhaustion, rendering them refractory to reactivation? Furthermore, the precise molecular mechanisms underpinning the differential pharmacological responses of many immune subsets to conventional chemotherapies and targeted agents remain poorly characterized. Dissecting how these drugs directly impact immune cell signaling pathways, metabolism, survival, and differentiation is crucial for predicting their immunomodulatory effects and rationally designing combinations. The complex crosstalk within the TME also warrants deeper investigation: how do non-immune components, like cancer-associated fibroblasts (CAFs) extensively discussed in the context of resistance, influence the pharmacological responsiveness of neighboring immune cells? How do TME-specific factors like hypoxia, nutrient availability, or metabolite accumulation shape immune subset function and drug sensitivity?

Addressing these questions is fundamental for developing robust predictive biomarkers. While PD-L1 expression and MSI status offer some guidance for CPIs, more refined biomarkers based on the pre-treatment composition, functional state, or spatial localization of specific immune subsets (e.g., CD8+/Treg ratio, specific TAM signatures, tertiary lymphoid structure maturity) are desperately needed to personalize therapy selection and predict patient outcomes more accurately.

Advancing our understanding in these areas necessitates the integration of cutting-edge multidisciplinary methodologies, as highlighted by the scope of this Research Topic. Single-cell multi-omics technologies (scRNA-seq, scATAC-seq, CITE-seq, single-cell proteomics) are indispensable for dissecting immune heterogeneity at unprecedented resolution, identifying novel subsets, defining their functional states, and mapping their responses to pharmacological perturbation. Spatial profiling techniques, such as multiplex immunofluorescence/immunohistochemistry, imaging mass cytometry, and spatial transcriptomics, are crucial for visualizing the intricate organization of immune subsets within the intact TME architecture, revealing critical cell-cell interactions and neighborhood effects that influence drug efficacy and immune function. Integrating these high-dimensional datasets requires sophisticated computational methods and systems biology approaches, including network analysis and agent-based modeling, to simulate immune population dynamics, predict therapeutic responses, and identify optimal strategies for combination therapies, potentially tailored to individual patient TME profiles. Furthermore, refining preclinical models—moving towards patient-derived organoids with immune components, humanized mouse models bearing patient TMEs, or syngeneic models more faithfully recapitulating human gastric cancer immunology—is essential for validating hypotheses and testing novel immunopharmacological strategies *in vivo*.

Within this framework, the future of nanoparticle-based immune subset targeting appears particularly bright, offering tangible tools to translate mechanistic understanding into therapeutic innovation. Future research will focus on refining NP design for enhanced *in vivo* specificity and delivery efficiency. This includes developing novel targeting ligands against more exclusive subset markers, engineering multi-stage delivery systems that sequentially overcome biological barriers (e.g., matrix penetration followed by cell targeting), and creating NPs that evade RES clearance more effectively. Advanced NP functionalities are also emerging, such as stimuli-responsive systems that release their payload only upon encountering specific TME cues (e.g., low pH, high enzyme activity, hypoxia), thereby increasing local drug concentration and reducing off-target effects. Theranostic nanoparticles, combining therapeutic delivery with imaging capabilities (e.g., using SPIONs or quantum dots), could enable non-invasive monitoring of NP biodistribution and potentially treatment response. Moreover, NPs provide an ideal platform for delivering next-generation immunomodulatory payloads, including mRNA for *in situ* vaccination or reprogramming of immune cells, siRNA for silencing immunosuppressive genes, or even CRISPR-based gene editing tools for durable modulation of immune function. While clinical translation faces hurdles related to safety evaluation (including potential immunogenicity of novel materials), scalable manufacturing, and navigating regulatory pathways, the potential benefits warrant concerted effort. Careful clinical trial design incorporating deep immune monitoring will be essential to validate the efficacy and safety of these promising nano-immunotherapies.

In conclusion, adopting an immunopharmacological perspective focused on immune subset diversity is a powerful paradigm shift for understanding and treating advanced gastric cancer, moving beyond tumor-centric approaches to embrace TME complexity. By integrating insights from advanced methodologies with innovative strategies like nanoparticle-based immune targeting, we can aspire to rationally modulate the tumor immune landscape. This approach holds the key to overcoming resistance, reinvigorating anti-tumor immunity, and paving the way for more effective, personalized treatments.
